# Marijuana Use in Inflammatory Bowel Disease: Understanding the Prevalence and the Potential Pitfalls

**DOI:** 10.1093/crocol/otaa016

**Published:** 2020-04-16

**Authors:** Frank I Scott

**Affiliations:** Crohn’s and Colitis Center, Division of Gastroenterology and Hepatology, University of Colorado Anschutz Medical Campus, Aurora, Colorado, USA

## Abstract

Many patients with inflammatory bowel disease experiment with marijuana for symptom control. Marijuana may help reduce abdominal pain but does not control inflammatory bowel disease related inflammation. Patients should discuss their marijuana use with their providers and continue appropriate medications if considering marijuana.

There has been significant interest in the potential therapeutic role of *Cannabis sativa*, also known as marijuana, in the treatment of inflammatory bowel disease (IBD).^1^ There are over 500 potentially active compounds within marijuana, with the two best studied compounds being cannabidiol (CBD) and Δ ^9^-tetrahydrocannibinol (THC). While the latter is felt to convey the vast majority of the psychoactive effects of marijuana, the former has been extensively studied for its effects on the endogenous endocannabinoid system. These compounds mitigate their effects through two main receptor families within the endocannabinoid system: CB1 receptors, which are found in the brain and enteric nervous system, and CB2 receptors, which are absent in the brain, but present in the enteric nervous system, gastrointestinal epithelial cells, and both macrophages and plasma cells. It is through interaction with both of these receptor classes that cannabinoids are felt to potentially modulate pain and inflammation.^1^

While murine models may suggest a direct effect of cannabinoids on inflammation in colitis models,^2^ the role of marijuana and its derivatives in human IBD remains uncertain. Initial observational uncontrolled studies demonstrated possible symptomatic improvement. In a case series of 30 patients conducted by Naftali et al,^3^ there was a noted reduction in Harvey Bradshaw Index scores and rates of corticosteroid use with active marijuana use. Similar results were seen in a prospective observational study of 13 patients with uncontrolled Crohn’s disease (CD) or ulcerative colitis prescribed 50 g marijuana daily. After 3 months of therapy, patients noted improved quality of life, reduced Harvey Bradshaw Index scores, and weight gain. However, there was no significant improvement in objective markers of inflammation such as C-reactive protein.^4^

This initial observational work has been followed by 2 small randomized controlled trials.^5, 6^ In the first of these studies, 21 patients with CD were randomized to either twice daily inhaled 115 mg THC or placebo.^5^ In this small study, 41% (5/11) of the treatment group achieved clinical remission, as defined by Crohn’s Disease Activity Index less than 150, compared to 10% (1 in 10) in the placebo group. All patients had recurrence of clinical symptoms after completing their 8-week course of cannabis. As with prior observational studies, there was no significant improvement in systemic markers of inflammation, such as CRP, leukocytosis, or hemoglobin. This was subsequently followed by another small randomized controlled trial of orally administered low dose CBD in 20 patients with CD.^6^ As opposed to their prior research using inhaled THC, there was no significant reduction in clinical symptoms with oral CBD. Based on these data, it appears that marijuana use may improve symptoms such as abdominal pain but may not impact the underlying inflammatory cascade in IBD.

In addition to the paucity of information on efficacy, there are also limited data on the prevalence of marijuana use among patients with IBD. The original research presented by Benson et al^7^ in this issue of *Crohn’s and Colitis 360* attempts to quantify the scope of marijuana utilization in Australia, as well as users’ perceptions regarding the possible benefits of its use. In a cross-sectional survey of 883 individuals who self-identified as having IBD, 25.3% reported prior or current use, and the vast majority of this was not via legal sources. About 18.1% reported they were actively using marijuana to manage their IBD symptoms, and frequency of use was nearly daily. Ninety-seven percent of patients with ulcerative colitis and 82% of patients with CD actively using marijuana felt it had a positive impact on IBD-related symptoms. Surveyed individuals felt that marijuana use had the greatest positive benefit on abdominal pain, stress, cramping, sleep-related issues, and anxiety. About half of active users also noted reductions in analgesic use related to their marijuana use.

The results presented by Benson et al are similar to those reported in the United States and Canada. For example, Allegretti et al^8^ measured self-reported marijuana use at a single academic medical center in Boston, Massachusetts. In this cross-sectional survey of 292 patients with IBD, 51.3% noted ever having used marijuana, with 12.3% reporting active use. Within this cohort, 16.4% noted they used marijuana for symptom control, with improvements in abdominal pain. Another cross-sectional study by Hoffenberg et al^9^ assessed marijuana use among pediatric patients with IBD; 32% of 99 participants had noted having ever used marijuana. While 9% of respondents reported daily or near-daily use, serum cannabinoids were detected in 50% of the 32 “ever” users. Approximately half of those reporting prior or current marijuana use noted they did so to manage abdominal pain. Hansen et al^10^ surveyed 201 patients with IBD in Winnipeg, Manitoba, Canada in 2018 and found higher marijuana use rates. In this study, 54% reported ever using marijuana, and 23% of those surveyed reported using marijuana for symptom control. Collectively, these findings confirm that a significant proportion of patients are experimenting with marijuana for IBD-related symptom control. As both medical and recreational marijuana legalization continues to expand, monitoring utilization rates is crucial for understanding prevalence and perceived benefits.

Benson et al^7^ also highlight some of the potential risks of marijuana use among patients with IBD. Despite the improvement in symptoms, marijuana users reported fewer interactions with gastroenterologists and reduced engagement with IBD specialists. Marijuana users also noted decreased medication adherence and increased rates of hospitalization in comparison to non-marijuana users. These outcomes should be interpreted with caution given the inability to assign causality or directionality to associations given the cross-sectional study design. However, these findings would be consistent with another prior retrospective cohort study that appreciated an increased risk of surgery in patients with CD with greater than 6 months of marijuana use compared to non-users (odds ratio 5.03, 95% confidence interval 1.45–17.46).^11^ If confirmed prospectively, these findings would suggest that additional counseling regarding medication adherence and follow-up may be required for patients with IBD who are using marijuana.

This and other studies have additionally demonstrated several well-described neuropsychiatric associations with chronic marijuana use. Benson et al^7^ noted increases in self-reported memory impairment, fatigue, and drowsiness. Among pediatric patients, Hoffenberg et al^9^ noted that 20% of patients reported regularly craving marijuana, and 13% noted neglecting responsibilities at home or at school due to marijuana use. In the previously mentioned Canadian study conducted by Hansen et al,^10^ depression and impulsivity were both increased in marijuana users, and an additional increased risk of substance misuse was appreciated. Given these collective findings, it would be appropriate to consider screening for depression, fatigue, and substance abuse when identifying marijuana use in our patient population.

Several caveats need to be considered when considering Benson’s research as well as the body of literature on marijuana use in IBD as a whole. First, the vast majority of available research is cross-sectional in nature, limiting the interpretability with regard to associations appreciated. Second, these studies rely on self-report for the calculation of prevalence rates. As noted by Hoffenberg’s study, in which cannabinoid levels were also measured, it is likely that there may be underreporting and subsequent exposure misclassification. Third, with multiple potential different modes of delivery and variation in cannabinoid concentrations, there is almost certainly significant heterogeneity in exposure. Last, the majority of studies to date rely on self-report for symptom improvement, which could introduce bias via outcome misclassification.

There may still be a role for marijuana in the IBD treatment paradigm, although considerable future research is much needed. While the slow wheels of science turn onward, this work by Benson et al demonstrates that a significant proportion of patients with IBD are turning to marijuana to manage their IBD-related symptoms. Additional prospective research to better quantify both the potential benefits in symptom reduction and any possible deleterious effects is required. While we await the discovery of additional information, we should be screening our patients for marijuana use. In those who are using or who are considering using marijuana, we should discuss continued use of appropriate, effective medical therapies to treat their IBD, ensure they have an appropriate follow-up, and screen for other comorbidities that may be associated with marijuana use.

## Notes

Disclosures: F.I.S. reports having grants from Takeda Pharmaceuticals USA, Janssen Pharmaceuticals, the National Institutes of Health, and the Crohn’s and Colitis Foundation. He has received consultant fees from PRIME Incorporated, Janssen Pharmaceuticals, Takeda Pharmaceuticals, and Merck Pharmaceuticals.
